# The Aging Microenvironment Shapes Angiogenic Remodeling in IBD‐Associated Colorectal Carcinogenesis

**DOI:** 10.1111/acel.70570

**Published:** 2026-06-14

**Authors:** Ruoshu Duan, Qingyu Chen, Yuan Xu, Ye Yan, Sujing Jiang

**Affiliations:** ^1^ Department of Gastroenterology The Second Affiliated Hospital and Yuying Children’s Hospital of Wenzhou Medical University Wenzhou Zhejiang P. R. China; ^2^ Department of General Practice The First Affiliated Hospital of Wenzhou Medical University Wenzhou Zhejiang P. R. China; ^3^ Department of Gastroenterology The First Affiliated Hospital of Wenzhou Medical University Wenzhou Zhejiang P. R. China

## Abstract

Chronic intestinal inflammation establishes a pro‐senescent microenvironment that fuels the stepwise evolution from inflammatory bowel disease (IBD) to colorectal cancer. Although cellular senescence initially functions as a tumor‐suppressive barrier, the persistent accumulation of senescent cells can promote disease progression through the senescence‐associated secretory phenotype (SASP). Key SASP mediators, including VEGF, IL‐8/CXCL8, and matrix metalloproteinases (MMPs), reprogram endothelial and stromal compartments, driving aberrant neovascularization, vascular leakiness, extracellular‐matrix remodeling, and tissue hypoxia that further reinforce inflammation and genomic instability. Emerging evidence also highlights marked heterogeneity among senescent epithelial, stromal, endothelial, and immune‐cell populations within the inflamed intestinal mucosa, suggesting that distinct senescent subsets may differentially shape angiogenesis and malignant transformation. This review synthesizes current evidence linking inflammation‐induced senescence to vascular dysfunction and the transition from IBD to colitis‐associated colorectal cancer, and discusses therapeutic opportunities targeting the senescence–angiogenesis axis. By clarifying how the aging microenvironment reshapes intestinal angiogenesis, we propose a mechanistic framework for early intervention and cancer prevention in colitis‐associated neoplasia.

## Graphical abstract

In IBD, chronic inflammation promotes cellular senescence across epithelial, stromal, endothelial, and immune compartments. The resulting SASP drives pathological angiogenesis, vascular dysfunction, hypoxia, and immune suppression, thereby facilitating progression from inflammation to dysplasia and colitis‐associated colorectal cancer. Targeting the senescence–angiogenesis axis may offer new opportunities for prevention and therapy.

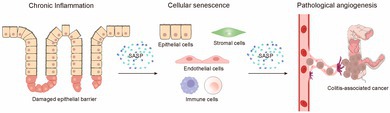

## 1 Introduction

Inflammatory bowel disease (IBD), encompassing Crohn's disease (CD) and ulcerative colitis (UC), is a chronic relapsing inflammatory disorder of the gastrointestinal tract that confers a significantly elevated risk of developing colorectal cancer (CRC) (Liu et al. 2024). The progression from persistent inflammation to colitis‐associated cancer (CAC) represents a classic example of inflammation‐driven oncogenesis, a multifaceted process orchestrated by dynamic interactions between immune cells, the epithelium, and the stromal microenvironment (Li et al. 2023). Although CAC shares certain molecular features with sporadic CRC, it differs in both its developmental trajectory and molecular evolution. While sporadic CRC typically follows the adenoma‐carcinoma sequence initiated by early APC mutations, followed by KRAS activation and later TP53 alterations, CAC arises through a distinct inflammation‐dysplasia‐carcinoma pathway (Carethers and Jung 2015). In CAC, TP53 abnormalities occur early and frequently, and may already be detected in chronically inflamed, non‐dysplastic mucosa, whereas APC mutations are less frequent and generally emerge at later stages of neoplastic progression; KRAS mutations also appear to be less common and tend to occur later in CAC than in sporadic CRC (Rajamäki et al. 2021). These features support the view that chronic mucosal inflammation imposes a distinct evolutionary pressure on intestinal tumorigenesis.

Beyond genetic alterations, the aging microenvironment has recently emerged as a critical determinant of cancer progression (Fane and Weeraratna 2020). Aging is one of the largest risk factors for cancer overall, a phenomenon attributed not only to time‐dependent accumulation of mutations but also to fundamental changes in the tissue microenvironment (López‐Otín et al. 2023). A cornerstone of this aged microenvironment is cellular senescence, a state of stable cell cycle arrest. Senescent cells accumulate with age and at sites of chronic pathology, where they secrete a plethora of bioactive factors known as SASP that can remodel the local tissue environment, influencing processes such as immune modulation, fibrosis, and notably, angiogenesis (Gorgoulis et al. 2019).

In this aging and chronically inflamed mucosal microenvironment, the efficacy of VEGF‐centered anti‐angiogenic strategies may also be limited, underscoring the need to better define the distinct angiogenic drivers and therapeutic vulnerabilities of CAC (Waldner et al. 2010). Rather than relying solely on VEGF, angiogenesis in CAC is likely sustained by a broader and more redundant network of pro‐angiogenic mediators induced by persistent inflammation, vascular remodeling, and tissue injury (Francescone et al. 2015). In this context, the sustained senescence–angiogenesis axis may be particularly relevant. Senescent stromal and endothelial cells, together with other chronically activated cellular components of the IBD microenvironment, can acquire a senescence‐associated secretory phenotype (SASP) and release multiple pro‐inflammatory and pro‐angiogenic mediators, including IL‐8, HGF, FGF‐family ligands, and matrix‐remodeling factors, thereby providing VEGF‐independent escape routes for pathological vascular remodeling (Coppé et al. 2010; Chambers et al. 2021). Such compensatory signaling may help maintain nutrient supply, inflammatory cell trafficking, and a tumor‐promoting niche even when VEGF signaling is partially inhibited, thereby facilitating malignant progression from chronic inflammation to dysplasia and cancer (Waldner et al. 2010). VEGF signaling is clearly involved in CAC biology, but experimental colitis‐associated cancer data also support the idea that angiogenesis in this setting is tightly intertwined with inflammatory signaling rather than governed by a single vascular axis alone.

**FIGURE 1:**
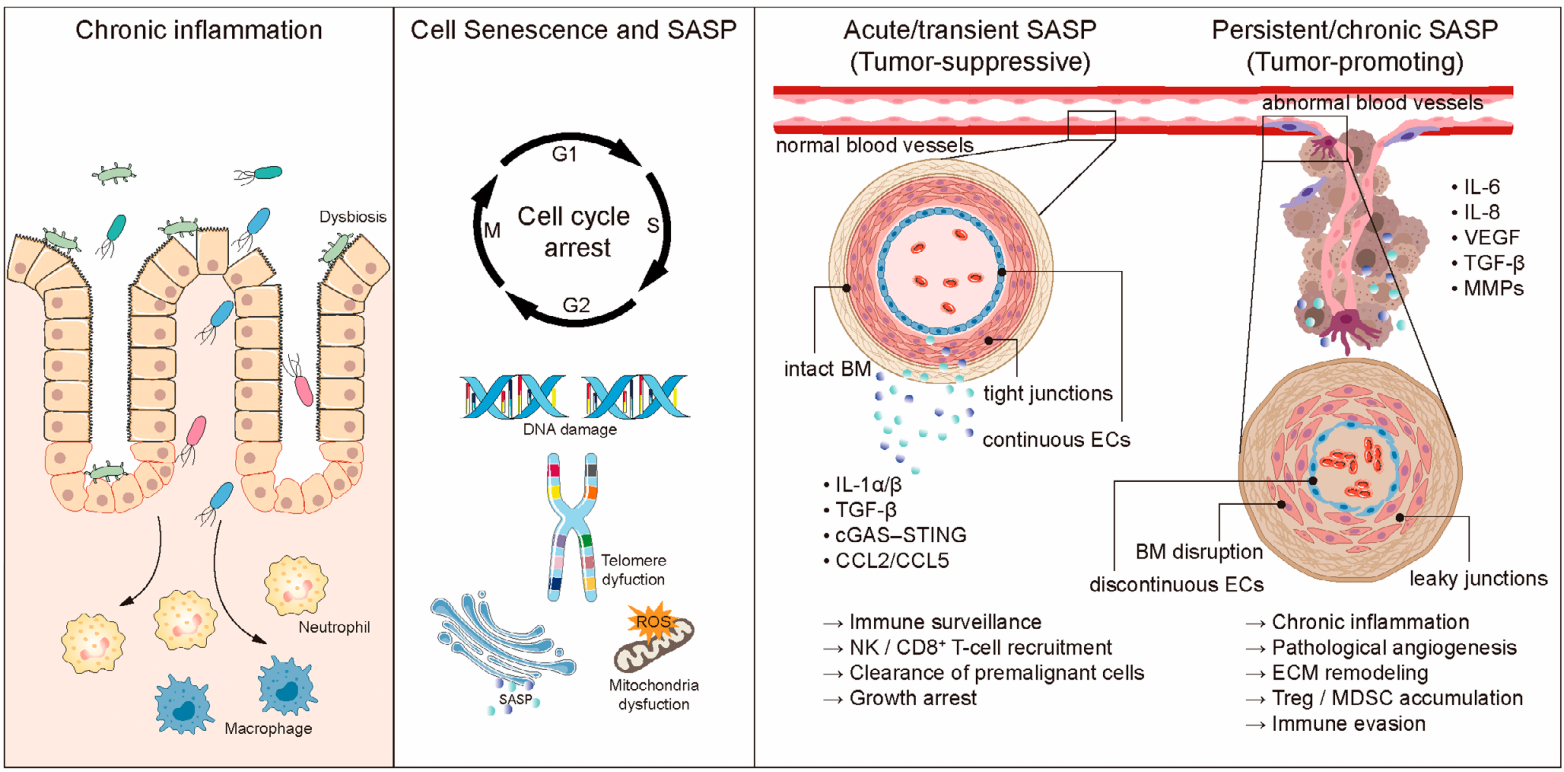
Dual role of senescence‐associated secretory phenotype (SASP) in angiogenic remodeling during IBD‐associated colorectal carcinogenesis. Chronic intestinal inflammation, driven by persistent mucosal injury, inflammatory cytokines, oxidative stress, and microbial stimulation, promotes the accumulation of senescent cells in the intestinal microenvironment. Senescent cells are characterized by stable cell‐cycle arrest, DNA damage and telomere dysfunction, mitochondrial ROS generation, and acquisition of a senescence‐associated secretory phenotype (SASP). In an acute/transient setting, SASP may exert tumor‐suppressive effects through mediators such as IL‐1α/β, TGF‐β, cGAS‐STING, and CCL2/CCL5, thereby promoting immune surveillance, recruitment of NK and CD8^+^ T cells, clearance of premalignant cells, and growth arrest. This state is associated with relatively normal vasculature, characterized by an intact basement membrane (BM), continuous endothelial cells, and tight intercellular junctions. In contrast, persistent/chronic SASP shifts toward a tumor‐promoting program enriched in IL‐6, IL‐8, VEGF, TGF‐β, and matrix metalloproteinases (MMPs), leading to chronic inflammation, extracellular‐matrix remodeling, pathological angiogenesis, accumulation of regulatory T cells (Tregs) and myeloid‐derived suppressor cells (MDSCs), and immune evasion. Pathological vessels display abnormal vascular architecture, BM disruption, discontinuous endothelial lining, and leaky junctions, thereby creating a hypoxic, inflamed, and tumor‐permissive microenvironment that supports dysplasia and colitis‐associated cancer.

Within this pro‐senescent and chronically inflamed setting, aberrant angiogenesis becomes a critical determinant of cancer progression, providing metabolic support for tumor growth and facilitating metastatic dissemination (Senga and Grose 2021). Yet, the contribution of senescence and SASP‐driven signaling to the dysregulated angiogenic response in CAC remains poorly understood. This review aims to fill this gap by critically examining how the aging microenvironment, driven by cellular senescence and the SASP, modulates tumor angiogenesis to promote CAC (Figure 1). We will explore the mechanistic underpinnings of this relationship and discuss the translational potential of targeting senescent cells in combination with anti‐angiogenic strategies for the prevention and treatment of IBD‐associated cancer.

## 2 The Inflammation‐Senescence‐Cancer Nexus in IBD

The “inflammation‐dysplasia‐carcinoma” sequence in IBD is not merely a passive background for tumorigenesis; rather, it constitutes an active driver that perpetuates tissue injury and aberrant repair. Persistent inflammatory stimuli, comprising pro‐inflammatory cytokines (e.g., TNF‐α, IL‐6, IL‐1β), reactive oxygen species (ROS), and microbial dysbiosis, continuously damage intestinal epithelial and stromal cells. This repetitive insult activates canonical signaling pathways such as NF‐κB and STAT3, which simultaneously sustain inflammation, induce epithelial hyperproliferation, and destabilize genomic integrity, thereby creating a permissive niche for carcinogenesis (Shahgoli et al. 2024). One critical cellular consequence of this inflammatory milieu is the induction of stress‐induced premature senescence (SIPS). Unlike telomere‐dependent replicative senescence, SIPS is rapidly triggered by oxidative stress, DNA damage, and persistent inflammatory signaling (Dierick et al. 2002). In IBD, both epithelial and stromal compartments exhibit features of senescence, including p16^INK4a^ and p21^CIP1^ expression, chromatin remodeling, and growth arrest (Luo et al. 2025). Initially, senescence protects against clonal expansion of damaged cells, but the persistent accumulation of senescent fibroblasts, mesenchymal stem cells, and immune cells ultimately remodels the mucosal microenvironment.

Senescent cells exert many of their pathological effects through the SASP. Factors such as IL‐8, VEGF, and MMPs amplify local inflammation, remodel the extracellular matrix, and stimulate pathological angiogenesis (Tufail et al. 2024). This pro‐angiogenic and pro‐inflammatory secretome establishes a feed‐forward loop that recruits immune cells, promotes immune evasion, and drives epithelial proliferation, collectively fostering the emergence of CAC. Notably, SASP composition evolves over time, shifting from an acute, wound‐healing response to a chronic, tumor‐promoting state when senescent cells persist.

The dual nature of senescence is increasingly recognized in the context of CAC. Early lesions experience a tumor‐suppressive effect, wherein senescence restricts proliferation of cells harboring oncogenic mutations. However, when senescent cells accumulate unchecked, they act as active participants in tumorigenesis. In acute or transient settings, senescence may function as an anti‐tumorigenic program by enforcing stable growth arrest and facilitating immune‐mediated clearance of damaged or premalignant cells. This early phase of senescence is associated with a context‐dependent secretory program that can promote immune surveillance and restrict clonal expansion. By contrast, when senescent cells persist and evade effective clearance, chronic SASP signaling may shift the intestinal microenvironment toward sustained inflammation, angiogenesis, extracellular‐matrix remodeling, and immune suppression, thereby promoting dysplasia and malignant progression. This functional transition is likely influenced by regulators such as NOTCH1/C/EBPβ, NF‐κB, mTOR, p38 MAPK, and cGAS‐STING‐associated signaling, which shape the timing, intensity, and inflammatory composition of SASP output (Cao et al. 2025). Preclinical studies support this context‐dependent role. In a murine model of colitis‐associated cancer, blockade of VEGFR2 signaling increased tumor‐cell senescence and reduced tumor burden, indicating that VEGFR2 signaling can restrain senescence in intestinal tumor cells, at least in part through the PI3K/AKT‐p21 pathway, and thereby promote tumor progression (Foersch et al. 2015). In addition, senescent mesenchymal stem cells have been shown to promote colorectal cancer cell growth through galectin‐3‐mediated signaling, supporting the broader concept that persistent senescent stromal‐like cells may contribute to a pro‐tumorigenic microenvironment (Li et al. 2015). Collectively, cellular senescence underscores a critical link between chronic inflammation and microenvironmental remodeling in CAC (Figure 1).

## 3 Molecular Mechanisms and Heterogeneity of the Senescent Cell Phenotype

### 3.1 Hallmarks of Senescence in IBD

Cellular senescence is a conserved stress response that enforces stable proliferative arrest while reprogramming cellular functions. In IBD, senescence is triggered not only by classical stressors such as DNA damage, telomere attrition, and oxidative stress, but also by persistent inflammatory signaling from dysregulated immune responses. Pro‐inflammatory monocytes and macrophages accumulate in the lamina propria, secreting cytokines including TNFα, IL‐6, and IL‐1β, which induce epithelial and stem cell stress and reinforce local senescence (Liao et al. 2024; Zhang, Shi, et al. 2025). Following López‐Otín et al., hallmarks of senescence are defined by three criteria: they manifest progressively over time, can be experimentally accelerated, and are amenable to therapeutic modulation. In IBD, these hallmarks span multiple levels: molecularly, activation of tumor‐suppressor pathways (p53/p21^CIP1^, p16^INK4a^/RB), DNA damage responses, telomere shortening, and chromatin reorganization such as senescence‐associated heterochromatin foci (SAHF) are observed; within cells, features include apoptosis resistance via BCL‐2 family proteins, metabolic remodeling, and loss of nuclear lamina components such as lamin B1; within tissues, persistent secretion of pro‐inflammatory mediators forms the SASP, which remodels the mucosal microenvironment, sustains chronic inflammation, and drives tissue remodeling and disease progression (López‐Otín et al. 2023).

### 3.2 Core Components of the SASP in IBD

The SASP is a principal mechanism by which senescent cells modulate tissue homeostasis and pathology in IBD. Rather than a uniform secretory program, the SASP comprises a diverse and context‐dependent collection of bioactive molecules that collectively shape the intestinal microenvironment, influencing both reparative and pathogenic processes. Key SASP components include pro‐inflammatory cytokines (e.g., IL‐6, IL‐8) that reinforce senescence and recruit immune cells; chemokines (e.g., CCL2, CXCL1) that guide leukocyte infiltration and organization; growth factors (e.g., TGF‐β, VEGF) that regulate tissue remodeling and angiogenesis; and matrix‐remodeling enzymes (e.g., MMPs) that alter extracellular matrix composition and epithelial barrier integrity. Beyond proteins, the SASP also encompasses bioactive lipids, extracellular vesicles containing proteins, lipids, and nucleic acids, and non‐coding RNAs or cytoplasmic chromatin fragments that can trigger stress responses in neighboring cells (Wang, Han, et al. 2024). Together, these factors generate a dynamic signaling milieu whose net effects depend on their duration, concentration, and tissue context. In IBD, a transient SASP can support wound repair and immune surveillance, whereas a persistent or dysregulated SASP drives chronic inflammation, fibrosis, and promotes neoplastic transformation. This functional diversity lays the foundation for understanding how cellular heterogeneity shapes the spatiotemporal dynamics of SASP‐mediated effects in the inflamed intestine.

## 4 Crosstalk Between Senescence, Inflammation, and Angiogenesis in IBD: The Dynamic Evolution From Inflammation to Cancer

### 4.1 Cellular Heterogeneity of Senescence in the Intestinal Microenvironment

Cellular senescence within the intestinal mucosa is a multifaceted and lineage‐specific process rather than a uniform endpoint. Single‐cell transcriptomic studies across mucosal and non‐mucosal tissues have demonstrated that senescent subsets defined by high expression of p16^INK4a^ or p21^CIP1^ occupy distinct niches and display unique secretory phenotypes, challenging single‐marker definitions of senescence (Mahmud et al. 2024). Recent studies further indicate that senescence‐associated programs in the intestinal microenvironment are distributed across multiple cellular compartments, with distinct functional consequences for inflammation, fibrosis, angiogenesis, and neoplastic evolution.

#### 4.1.1 Epithelial Cells

In the inflamed intestine, sustained exposure to luminal antigens and reactive oxygen species imposes genotoxic stress that drives epithelial cells into diverse senescent states (Brandt et al. 2022). These senescence‐like epithelial states are associated with disturbed arginine‐NO metabolism, regenerative stress, and a secretome enriched in cytokines and matrix metalloproteinases that disrupt junctional complexes and heighten mucosal permeability. Functionally, epithelial senescence contributes primarily to barrier dysfunction and persistent mucosal injury. In addition, senescent epithelial cells may contribute to a pro‐angiogenic SASP milieu through the release of mediators such as VEGF and IL‐8/CXCL8, thereby linking epithelial injury to aberrant vascular remodeling and a tumor‐permissive microenvironment (Jeong et al. 2021; Gabryel et al. 2025).

#### 4.1.2 Stromal Components

Stromal senescence represents a second major compartment contributing to tissue remodeling in IBD and CAC. Compared with epithelial senescence, stromal senescence is more closely associated with extracellular‐matrix reorganization, fibrosis, vascular instability, and chronic inflammatory persistence.

##### 4.1.2.1 Fibroblasts

Senescence‐associated fibroblast populations are strongly linked to extracellular‐matrix remodeling, fibrosis, and maintenance of the chronic inflammatory niche (Cadinu et al. 2024; Rieder et al. 2024). In the inflamed mucosa, fibroblast subsets develop profibrotic and immune‐interacting phenotypes enriched in TGF‐β and extracellular matrix proteins, thereby remodeling the mucosal scaffold, promoting stricture formation, and increasing tissue rigidity (Korsunsky et al. 2022; Kinchen et al. 2018).

##### 4.1.2.2 Pericytes

Pericytes within the perivascular niche may undergo age‐ or senescence‐associated dysfunction that impairs vascular support. These changes are expected to weaken endothelial‐pericyte interactions, compromise vessel stabilization, and promote abnormal vessel maturation, thereby contributing to vascular leakiness and pathological angiogenesis (Geevarghese and Herman 2014; Armulik et al. 2005).

##### 4.1.2.3 Endothelial Cells

Endothelial senescence‐like programs directly link microenvironmental aging to vascular dysfunction (Bloom et al. 2023). Senescent endothelial cells may exhibit impaired barrier properties, aberrant activation, and altered secretory profiles that favor vessel immaturity, increased permeability, and maladaptive neovascularization. Through these effects, endothelial senescence becomes a key mediator of angiogenic remodeling in the chronically inflamed intestine.

#### 4.1.3 Immune Cells

Senescence‐ and aging‐associated changes are also evident in immune compartments, including effector and regulatory T cells as well as monocyte/macrophage populations. These maladaptively aged immune subsets display altered functional properties that can reinforce chronic inflammation rather than resolve it. In particular, aging‐associated monocyte/macrophage programs may contribute to a low‐grade pro‐inflammatory state and sustained cytokine production, potentially reinforcing NF‐κB‐related inflammatory circuits and sustaining a pro‐inflammatory, pro‐angiogenic microenvironment (Moss et al. 2023; Tilstra et al. 2011), thereby sustaining the inflammatory and angiogenic milieu. Collectively, these findings support the view that senescence in IBD and CAC is a multicellular and functionally heterogeneous process, in which epithelial, stromal, endothelial, and immune compartments contribute in distinct yet interconnected ways to disease progression.

### 4.2 From Chronic Inflammation to Cellular Senescence

In the chronically inflamed intestine, persistent cytokine exposure, oxidative stress, and repeated tissue injury promote the emergence of senescent cell states across multiple cellular compartments. Having outlined the multicellular heterogeneity of senescence in the intestinal microenvironment, we next consider how chronic inflammatory stress drives these senescence‐associated states across epithelial, stromal, and immune compartments. In the chronically inflamed intestine, persistent cytokine exposure, oxidative stress, and repeated tissue injury promote the emergence of senescent cell states across multiple cellular compartments.

#### 4.2.1 Epithelial Compartment

Mechanistically, chronic intestinal inflammation accelerates the epithelial senescence programs described above by establishing a biochemical environment enriched in pro‐inflammatory cytokines, oxidative stress, and repeated injury‐repair cycles. Sustained exposure to pro‐inflammatory cytokines, including TNF‐α and IL‐6, drives NADPH oxidase‐mediated ROS accumulation and mitochondrial dysfunction, resulting in DNA damage and activation of the ATM/ATR‐mediated DNA damage response, with downstream engagement of p53/p21 and p16^INK4a^‐dependent checkpoints (Coppé et al. 2010). Persistent signaling through these pathways promotes stable cell‐cycle arrest and the development of a SASP, thereby contributing to epithelial dysfunction and impaired mucosal repair. In murine colitis models, prolonged inflammatory stimulation induces p16 and p21 expression in intestinal epithelial crypts, accompanied by telomere attrition, compromised barrier integrity, and distorted crypt architecture (Gan et al. 2026; Lopetuso et al. 2025). Interventions targeting inflammation or oxidative stress, such as COX‐2 inhibition or lactoferrin treatment, partially attenuate p16/p21 induction and restore crypt morphology, supporting a causal link between chronic cytokine exposure, senescence induction, and structural epithelial injury (Lopetuso et al. 2025; Sienkiewicz et al. 2023).

#### 4.2.2 Stromal Compartment

In stromal populations, chronic inflammatory and genotoxic stress mechanistically drives the senescence‐associated remodeling outlined above, particularly in fibroblasts, mesenchymal cells, and endothelial cells. Under sustained inflammatory and genotoxic stress, these cells may acquire senescence‐associated programs characterized by altered extracellular‐matrix production, profibrotic signaling, and aberrant intercellular communication (Rieder et al. 2024). Such stromal remodeling may promote matrix deposition, tissue stiffness, maladaptive repair, and vascular dysfunction, thereby creating a microenvironment that supports persistent inflammation and pathological angiogenesis. In particular, fibroblast‐driven extracellular‐matrix remodeling is increasingly recognized as a central determinant of intestinal fibrosis in IBD, while endothelial dysfunction contributes to abnormal vascular responses in chronically inflamed tissue (Wei et al. 2025; Zhang, Zhuang, et al. 2025).

#### 4.2.3 Immune Compartment

In immune cells, persistent antigenic and inflammatory stimulation promotes the senescence‐like and dysfunctional states introduced above, thereby progressively reshaping mucosal immune surveillance in IBD (Fu et al. 2025). T cells exposed to persistent antigenic stimulation progressively acquire a highly differentiated immunosenescent phenotype characterized by loss of CD28 and CD27 expression, telomere shortening, and upregulation of markers such as KLRG1 and CD57 (Yang and Yuan 2025; Shen et al. 2026). In parallel, exhausted T‐cell states arise under continuous antigen exposure and are associated with impaired effector function and reduced immune control (Wu et al. 2026). Myeloid populations are likewise altered: monocytes and macrophages exposed to sustained inflammatory cues may develop senescence‐associated or maladaptive activation programs marked by persistent NF‐κB signaling, increased secretion of IL‐1β, IL‐6, and TNF, and reduced phagocytic/efferocytic capacity (Liao et al. 2024; Zhang, Shi, et al. 2025; Chen et al. 2025; Wang, Hong, et al. 2024). This decline in immune‐cell fitness may limit the efficient clearance of damaged and senescent cells, thereby reinforcing SASP persistence and chronic inflammatory signaling within the intestinal mucosa. Consistent with this, patients with long‐standing IBD exhibit features of premature immunosenescence, including reduced T‐cell receptor diversity, altered proliferative capacity, and disturbed mucosal immune homeostasis (Shen et al. 2026; Mahdy et al. 2026). Collectively, these findings support the view that chronic intestinal inflammation acts as an upstream driver of senescence across epithelial, stromal, and immune compartments, thereby sustaining mucosal dysfunction and priming a microenvironment that favors pathological remodeling and increased cancer risk.

### 4.3 From Cellular Senescence to Angiogenesis

Once established across multiple cellular compartments, senescent states exert their downstream effects primarily through the SASP, which acts as the major effector linking chronic inflammation to angiogenic remodeling. The SASP serves as a pivotal conduit through which senescent cells remodel the intestinal microenvironment and drive the progression from chronic inflammation to colitis‐associated carcinogenesis in inflammatory bowel disease. By persistently releasing pro‐inflammatory cytokines, growth factors, proteolytic enzymes, and extracellular vesicles, the SASP undermines epithelial barrier integrity, amplifies mucosal inflammation, and activates angiogenic pathways, thereby creating a self‐reinforcing loop of tissue injury and maladaptive repair. Central to this process are pro‐inflammatory and matrix‐remodeling mediators, including IL‐1α/β, IL‐6, IL‐8, and matrix metalloproteinases, whose cell type‐specific secretion patterns orchestrate immune‐cell recruitment, extracellular matrix dynamics, and angiogenic activation within the inflamed intestine. IL‐1α and IL‐1β are recognized as pivotal upstream regulators of the senescence‐associated inflammatory network, acting through activation of the NF‐κB pathway to enhance the production of downstream SASP cytokines, notably IL‐6 and IL‐8, in epithelial and stromal contexts (Klepacki et al. 2025). While cell surface‐bound IL‐1α has been shown to be essential for NF‐κB‐driven IL‐6/IL‐8 secretion in senescent fibroblasts, accumulating evidence also implicates IL‐1β in amplifying intestinal inflammation and barrier disruption in IBD, suggesting that a similar IL‐1‐NF‐κB axis may operate in senescent intestinal cells and warrants further validation (Orjalo et al. 2009; Aggeletopoulou et al. 2024). In addition, NF‐κB and STAT3 signaling appear to form a reinforcing inflammatory–angiogenic circuit in endothelial and stromal compartments. Sustained NF‐κB activation promotes IL‐6 and IL‐8 production, whereas IL‐6‐driven JAK/STAT3 signaling further enhances VEGF and MMP‐9 expression, thereby coupling persistent inflammatory stress to endothelial activation, extracellular‐matrix remodeling, and pathological angiogenesis (Li et al. 2025; Mihăiluță et al. 2025). This signaling loop may also destabilize endothelial‐pericyte interactions, contributing to vessel immaturity, leakiness, and aberrant neovascularization.

Within the inflamed intestinal mucosa of IBD patients, pro‐inflammatory cytokines orchestrate angiogenic remodeling. IL‐1β acts as a potent driver by inducing VEGF and VEGFR2 expression on endothelial cells, thereby promoting neovascularization (Li et al. 2025). Simultaneously, elevated IL‐6 levels in both UC and CD activate the JAK/STAT3 signaling pathway, enhancing the transcription of VEGF and MMP‐9, which collectively stimulate endothelial proliferation, migration, and capillary network formation (Shahini and Shahini 2023; Calviño‐Suárez et al. 2025). These cytokine‐mediated cascades establish a mechanistic link between sustained mucosal inflammation and vascular remodeling, providing a permissive microenvironment for inflammation‐associated tumor progression. Beyond these well‐characterized cytokine‐mediated pathways, additional soluble mediators further contribute to the angiogenic landscape in IBD. IL‐8, abundant in inflamed IBD lesions, acts as both a chemoattractant and mitogen via CXCR1/2 engagement, while IL‐10, despite its canonical anti‐inflammatory function, can indirectly enhance angiogenesis by polarizing macrophages toward pro‐angiogenic phenotypes and dampening immune surveillance (Sokólska et al. 2026; Tufail et al. 2025).

Beyond cytokine signaling, proteolytic components of the SASP further exacerbate this remodeling. MMP‐2, MMP‐9, and MMP‐13, together with the urokinase plasminogen activator (uPA) system, degrade basement membranes and remodel the extracellular matrix (ECM), liberating sequestered mediators such as VEGF, fibroblast growth factor 2 (FGF‐2), and TGF‐β (He et al. 2022; Boaru et al. 2025; Hamada et al. 2024). In IBD, ECM degradation establishes permissive channels for endothelial infiltration and amplifies pro‐angiogenic cues through the release of otherwise sequestered mediators. The ensuing cycles of matrix deposition and breakdown progressively reshape tissue mechanics, fostering vascular fragility and stimulating new sprout formation (Petrey and de la Motte 2017). Recurrent inflammation, epithelial injury, and maladaptive repair progressively lock the IBD mucosa into a self‐sustaining cycle. Through their senescence‐associated secretory phenotype, senescent cells remodel the extracellular matrix and drive aberrant angiogenesis; in turn, the restructured stroma and abnormal vasculature perpetuate inflammation and expand the senescent cell population. This reciprocal circuitry positions the SASP as a central orchestrator of microenvironmental remodeling and a key mediator linking chronic inflammation to pathological angiogenesis in IBD.

### 4.4 Angiogenesis as a Gateway to Dysplasia and CAC

As a downstream consequence of sustained SASP‐driven vascular remodeling, aberrant angiogenesis may function as a critical transition point linking chronic inflammation and senescence to dysplasia and CAC. Aberrant angiogenesis is a critical downstream consequence of the sustained crosstalk among chronic inflammation, cellular senescence, and SASP signaling in IBD, and may represent a key transition point toward dysplasia and CAC. Long‐standing IBD is characterized by tortuous, immature, and hyperpermeable neovessels that exacerbate mucosal hypoxia, oxidative stress, and metabolic instability (Britzen‐Laurent et al. 2023). Hypoxic conditions, particularly in ulcerated regions, stabilize HIF‐1α in intestinal tissues and promote the expression of VEGF and other pro‐angiogenic mediators, thereby sustaining a self‐reinforcing cycle of pathological vascular remodeling and expansion (Hou et al. 2024; Glover and Colgan 2011).

Within this chronically inflamed milieu, endothelial and stromal senescence further amplifies vascular dysfunction. Senescent endothelial cells (ECs), despite their reduced proliferative capacity, secrete pro‐inflammatory mediators such as CXCL1, which can act in a paracrine manner to activate neighboring ECs and promote vascular remodeling in inflamed tissue (Rolas et al. 2024). Endothelial senescence in IBD is unlikely to occur uniformly throughout the mucosa, but may instead arise preferentially in chronically inflamed, hypoxic, and ulcerated regions, where endothelial cells are exposed to persistent cytokine signaling, oxidative stress, and repeated injury‐repair cycles (Gravina et al. 2018). In this context, focal senescent ECs may propagate dysfunction to neighboring endothelial cells through SASP‐mediated paracrine or bystander‐like signaling, creating a patchy pattern of endothelial dysfunction that may resemble a “salt‐and‐pepper”‐like distribution of senescence‐associated changes in the inflamed mucosa (da Silva et al. 2019; Nelson et al. 2012). Although direct spatial evidence for this pattern in intestinal mucosa remains limited, it provides a plausible mechanistic framework for understanding how local endothelial senescence may drive vascular instability and immune‐cell recruitment in the IBD/CAC microenvironment. At the same time, endothelial populations within dysplastic regions may preserve angiogenic activity, allowing continued vascular support for neoplastic expansion (Dudley and Griffioen 2023). In parallel, the senescence‐associated microenvironment, including SASP‐derived mediators and senescent stromal cells, may further support dysplastic epithelial growth and survival (Choi et al. 2023).

**FIGURE 2:**
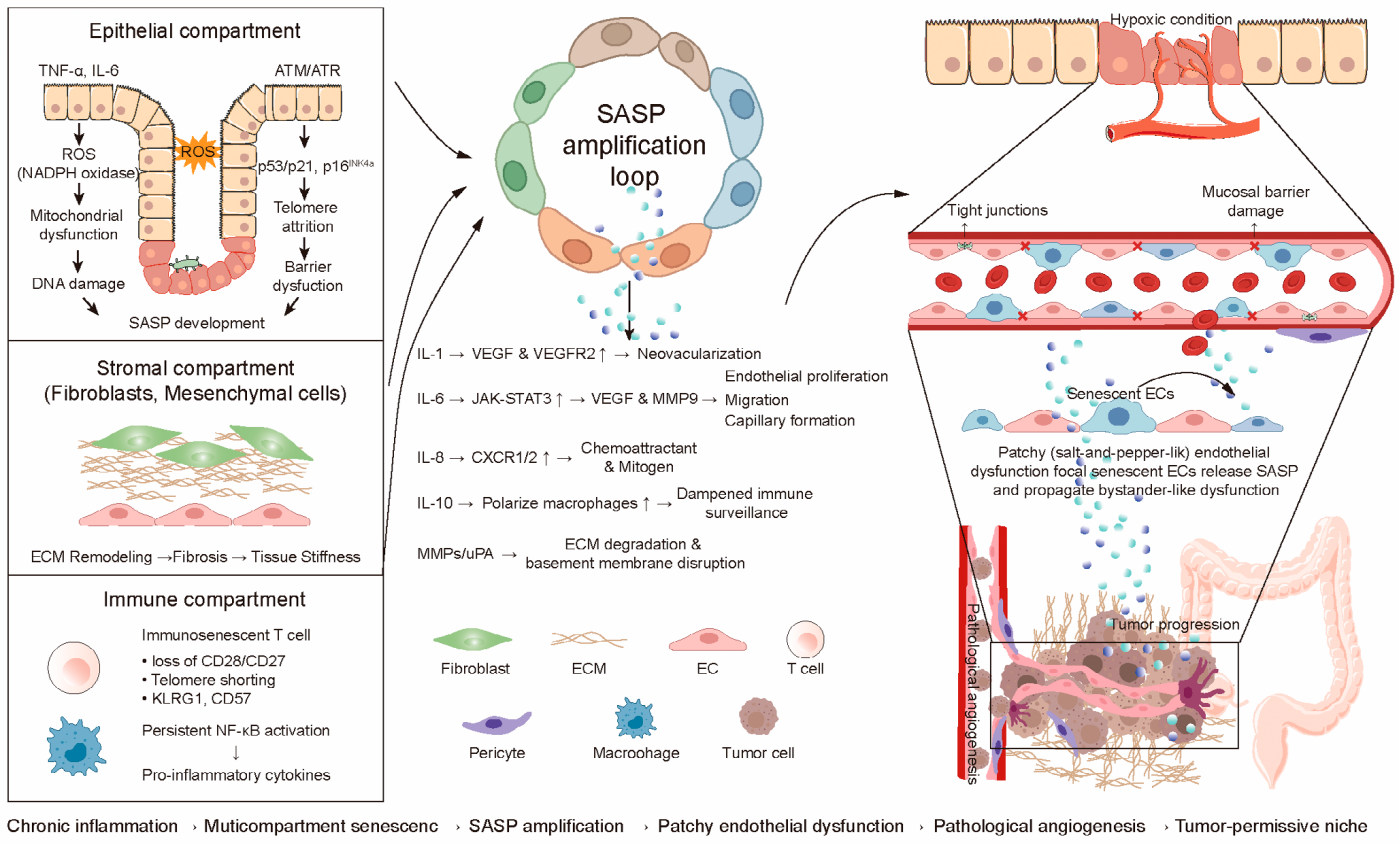
Zoomed‐in mechanistic view of the crosstalk among chronic inflammation, cellular senescence, and angiogenic remodeling in IBD. Persistent inflammatory stress and repeated tissue injury induce senescence‐associated programs across multiple cellular compartments in the intestinal mucosa. In the epithelial compartment, ROS accumulation, oxidative stress, and repetitive injury promote epithelial dysfunction and senescence. In the stromal compartment, fibroblasts and mesenchymal cells contribute to extracellular‐matrix remodeling, fibrosis, and tissue stiffening. In the immune compartment, immunosenescent T cells and chronically activated myeloid cells sustain NF‐κB‐driven inflammatory cytokine production. These signals converge in a SASP amplification loop involving mediators such as IL‐1, IL‐6, TNF, VEGFR2, and HIF‐1α. Downstream, IL‐1 enhances VEGF/VEGFR2‐associated angiogenic signaling, IL‐6 activates JAK/STAT3 and increases VEGF and MMP‐9 expression, IL‐8 acts through CXCR1/2, and IL‐10 may indirectly support angiogenic remodeling by promoting macrophage polarization toward pro‐angiogenic phenotypes. Together, these pathways promote endothelial activation, endothelial proliferation and migration, capillary formation, extracellular‐matrix remodeling, and pathological neovascularization. The schematic also illustrates focal endothelial dysfunction in inflamed and hypoxic mucosal regions, together with a patchy “salt‐and‐pepper‐like” propagation of senescence‐associated changes to neighboring endothelial cells through paracrine signaling. Collectively, these reciprocal interactions drive vascular leakiness, immune dysregulation, and formation of a tumor‐permissive niche that facilitates progression from chronic inflammation to colitis‐associated cancer.

Pathological angiogenesis in IBD may also contribute to the formation of an immunosuppressive niche. These pathological vessels are typically structurally immature, hyperpermeable, and persistently activated, with discontinuous endothelial junctions, abnormal barrier function, and reduced mural‐cell support, all of which contribute to vascular instability and disordered leukocyte trafficking (Britzen‐Laurent et al. 2023; Dudley and Griffioen 2023). In addition to sustaining hypoxia and inflammatory signaling, such vascular abnormalities may alter endothelial adhesion and chemokine cues, thereby helping establish an immunosuppressive niche that favors the accumulation of regulatory T cells (Tregs) and myeloid‐derived suppressor cells (MDSCs) while limiting effective cytotoxic T‐cell infiltration (Schaaf et al. 2018; He et al. 2025). Specifically, pathological angiogenesis is associated with downregulation of intercellular adhesion molecule‐1 (ICAM‐1) and vascular cell adhesion molecule‐1 (VCAM‐1) on endothelial cells, which reduces the adhesion and transendothelial migration of cytotoxic CD8^+^ T cells. At the same time, altered endothelial activation may favor the recruitment of immunosuppressive leukocyte subsets through selective expression of adhesion‐related molecules and a chemokine milieu enriched in CCL2, CCL5, and CXCL12, which support the accumulation of MDSCs and Tregs via CCR2, CCR5, and CXCR4 signaling, respectively. By contrast, reduced CXCL9/CXCL10‐mediated gradients may limit the recruitment of effector cytotoxic T cells. Together, these adhesion and chemokine changes contribute to an immunosuppressive vascular niche that preferentially supports Treg and MDSC enrichment. With persistent SASP signaling and defective immune clearance, this permissive vascular niche may facilitate immune evasion and progression toward malignancy. In parallel, senescent macrophages with impaired clearance capacity may further amplify SASP accumulation and chronic inflammatory signaling, thereby reinforcing this immunosuppressive vascular niche (Wang, Hong, et al. 2024). These changes enable genetically altered epithelial clones to evade immune surveillance and progress toward malignancy. Collectively, these observations support the view that pathological angiogenesis is not merely a by‐product of inflammation in IBD, but an active driver of microenvironmental remodeling that links chronic inflammation and senescence to dysplasia and CAC, as summarized in Figure 2.

## 5 Therapeutic Implications and Future Perspectives in IBD‐Associated Cancer

Senescent cells within the intestinal microenvironment contribute to tumor angiogenesis primarily through the secretion of SASP factors, including VEGF, IL‐6, IL‐8, CXCLs, and MMPs, which stimulate endothelial proliferation, migration, and capillary formation. Therapeutic strategies targeting these senescent cells, particularly senolytics and senomorphics, offer potential avenues to attenuate SASP‐mediated angiogenic signaling (Table 1). Representative senolytics, such as navitoclax (ABT‐263), venetoclax (ABT‐199), quercetin, fisetin, and HSP90 inhibitors (17‐DMAG, geldanamycin), act by selectively eliminating senescent cells through inhibition of anti‐apoptotic pathways, interference with PI3K/AKT signaling, and disruption of chaperone‐mediated survival mechanisms (Chaib et al. 2022). This targeted removal may indirectly reduce the secretion of pro‐angiogenic SASP factors, thereby potentially mitigating endothelial activation and neovascularization. In parallel, senomorphics, including rapamycin, AZD8055, metformin, resveratrol, ruxolitinib, and FGF21, modulate senescence and/or the SASP through pathways including mTOR, NF‐κB, AMPK, JAK/STAT, and SIRT1‐associated signaling. Unlike senolytics, senomorphics diminish the production of pro‐inflammatory and pro‐angiogenic factors without necessarily inducing cell death, thereby preserving tissue integrity while mitigating the deleterious paracrine effects of senescent cells (Han and Kim 2023).

**TABLE 1:** Representative senescence‐targeting agents and strategies reported to modulate senescence and/or the SASP, with potential relevance to angiogenesis in IBD‐associated colorectal cancer.

Type	Drug	Main targets/pathway	Mechanism of action	References
Senolytic	Navitoclax (ABT‐263)	BCL‐2, BCL‐xL, BCL‐w	Induces apoptosis of senescent cells by inhibiting anti‐apoptotic BCL‐2 family proteins	(Zhu et al. 2016)
Senolytic	Venetoclax (ABT‐199)	BCL‐2	Selectively induces apoptosis in BCL‐2‐dependent senescent cells	(As Sobeai et al. 2022)
Senolytic	A1331852	BCL‐xL	Highly selective BCL‐xL inhibitor inducing apoptosis in senescent fibroblasts and tumor cells resistant to ABT‐263.	(Zhu et al. 2017)
Senolytic	A1155463	BCL‐xL	Potently targets BCL‐xL to trigger intrinsic apoptotic signaling in senescent cells, with minimal effect on proliferating counterparts.	(Zhu et al. 2017)
Senolytic	Fisetin	PI3K, BCL‐xL	Exhibits broad senolytic activity by inhibiting PI3K and BCL‐xL; reduces SASP factors and mitigates therapy‐induced senescence	(Tavenier et al. 2024)
Senolytic	Quercetin	PI3K, BCL‐2	Suppresses PI3K/AKT signaling and downregulates anti‐apoptotic pathways, leading to selective clearance of senescent cells and SASP attenuation.	(Özsoy et al. 2020)
Senolytic	Dasatinib	Tyrosine kinase inhibition	Inhibits pro‐survival kinase signaling and induces senescent cell apoptosis, particularly in adipose and endothelial lineages	(Zhu et al. 2015)
Senolytic	HSP90 inhibitors (17‐DMAG, 17‐AAG, Geldanamycin)	HSP90	Blocks HSP90‐mediated stabilization of client proteins, essentially for senescent cell survival and promoting apoptosis	(Fuhrmann‐Stroissnigg et al. 2017)
Senolytic	Curcumin	NF‐κB, JAK/STAT	Modulates pro‐survival transcriptional programs and enhances senescent cell death; exerts additional anti‐inflammatory effects.	(Zia et al. 2021)
Senolytic	Piperlongumine	ROS signaling, GSTP1	Elevates intracellular ROS levels and inhibits glutathione S‐transferase, selectively inducing oxidative death in senescent cells.	(Liu et al. 2018)
Senolytic	FOXO4‐related peptide (FOXO4‐DRI)	p53‐FOXO4 interaction	Disrupts the interaction between FOXO4 and p53, releasing p53 from the nucleus and triggering apoptosis specifically in senescent cells.	(Li et al. 2024)
Senolytic	Fenofibrate	PPARα	Enhances autophagic flux and promotes selective clearance of senescent cells, indicating potential for age‐related degenerative disease intervention.	(Nogueira‐Recalde et al. 2019)
Senolytic (Cell‐based)	CAR‐T/NK Cell Therapy	uPAR	Engineered CAR‐T cells targeting uPAR selectively eliminate senescent cells in preclinical models.	(Amor et al. 2020)
Senomorphic	Rapamycin	mTOR	Inhibits mTOR‐dependent SASP regulation, including IL‐1α‐linked inflammatory output, thereby reducing pro‐inflammatory and pro‐angiogenic mediators and promoting a senomorphic state.	(Laberge et al. 2015; Herranz et al. 2015)
Senomorphic	AZD8055	mTOR	A dual mTORC1/2 inhibitor with potential senomorphic activity through suppression of mTOR‐dependent inflammatory/SASP signaling.	(Laberge et al. 2015; Herranz et al. 2015; Marshall et al. 2011)
Senomorphic	Metformin	AMPK/mTOR, NF‐κB	Attenuates the senescence‐associated secretory phenotype by interfering with IKK/NF‐κB signaling and improving metabolic stress responses.	(Moiseeva et al. 2013)
Senomorphic	Resveratrol	NF‐κB, SIRT1	Suppresses NF‐κB‐associated inflammatory/SASP signaling and activates SIRT1, thereby improving redox balance, stress responses, and senescence‐associated endothelial dysfunction.	(Kao et al. 2010; Xia et al. 2017)
Senomorphic	Ruxolitinib	JAK1/2	Inhibits the JAK–STAT pathway to block SASP cytokine secretion (IL‐6, IL‐8) and ameliorate senescence‐associated inflammation. Shown to reduce tissue fibrosis and endothelial activation in preclinical models.	(Yang et al. 2024)
Senomorphic	FGF21	SIRT1 activation	Endocrine regulator that activates SIRT1 signaling to attenuate NF‐κB‐driven SASP and oxidative stress. Promotes mitochondrial homeostasis and delays cellular senescence in metabolic and inflammatory disorders.	(Lu et al. 2021)

*Note:* Most evidence summarized in this table is derived from preclinical studies and, in several cases, from non‐IBD settings; direct evidence in IBD‐associated colorectal carcinogenesis remains limited.

Although most available evidence remains preclinical and is often derived from non‐IBD settings, mechanistic and experimental studies suggest that senescence‐targeting interventions may reduce pathological vascular remodeling, improve endothelial function, and enhance anti‐tumor immune surveillance in selected models. Importantly, the biological consequences of senescence are highly context dependent. The tumor‐promoting effects discussed in earlier sections mainly refer to the persistent accumulation of senescent stromal, endothelial, and immune cells together with a chronic SASP‐rich microenvironment. By contrast, acute or therapy‐induced senescence in established tumor cells may exert tumor‐suppressive effects by enforcing growth arrest and increasing susceptibility to immune‐mediated clearance. In murine CAC models, VEGFR2 inhibition induces tumor‐cell senescence through a PI3K/AKT‐p21‐dependent mechanism and is associated with reduced tumor burden and enhanced CD8+ T‐cell‐mediated cytotoxicity. Similarly, inhibition of VEGF signaling has been reported to induce senescence in colorectal cancer cells (Foersch et al. 2015; Hasan et al. 2011).

These findings support the potential translational application of senotherapeutics in IBD‐associated carcinogenesis. Strategic combination approaches may integrate senolytics or senomorphics with anti‐inflammatory biologics targeting TNF or IL‐23 pathways to reduce epithelial injury and SASP‐driven inflammation, or with anti‐VEGF/VEGFR‐directed agents to reinforce angiogenesis blockade (Table 2). Moreover, modulation of SASP factors may enhance responsiveness to chemotherapy and immunotherapy by promoting vascular normalization and restoring cytotoxic T‐cell function. Future investigations should prioritize the identification of robust senescence biomarkers, optimization of dosing regimens, and systematic evaluation of safety and efficacy in IBD‐associated tumorigenesis.

**TABLE 2:** Selected pharmacological modulators of angiogenesis and senescence with potential relevance to IBD‐associated colorectal cancer.

Type	Drug	Main targets/pathway	Effect on angiogenesis	Evidence for senescence modulation
Anti‐VEGF agents	Bevacizumab	VEGF‐A	Potent VEGF blockade to reduced pathological neovascularization (Hurwitz et al. 2004)	Induction (tumor‐cell senescence)‐direct translational evidence. VEGFR2/VEGF blockade induced senescence in CRC/CAC models and correlated with increased senescent cell fraction after treatment; senescence associated with immune effects (Hasan et al. 2011)
Anti‐VEGF agents	Aflibercept	VEGF‐A/B, PlGF	Suppresses neovascularization; potential endothelial stress (Ricci et al. 2015)	Unclear
Anti‐VEGF agents	Ramucirumab	VEGFR‐2	Blocks VEGFR2‐mediated angiogenic signaling (Tabernero et al. 2015)	Indirect/Limited. VEGFR2 blockade mechanistically intersects senescence pathways (VEGFR2 → PI3K/AKT → p21) but direct ramucirumab‐specific senescence data are scarce (Foersch et al. 2015)
Anti‐inflammatory biologics	Infliximab	TNF‐α	Reduces inflammation‐driven angiogenesis and oxidative stress (Rutella et al. 2011)	Indirect (reduces inflammation‐driven senescence/SASP). Anti‐TNF agents have been shown to modulate SASP‐related markers and to attenuate inflammation‐driven senescence phenotypes (Prattichizzo et al. 2016)
Anti‐inflammatory biologics	Adalimumab	TNF‐α	Reduces inflammation‐driven angiogenesis (Liu et al. 2015)	As above
Anti‐inflammatory biologics	Guselkumab	IL‐23 p19	Downregulates inflammatory angiogenic cytokines (Guo et al. 2023)	Very limited/indirect. IL‐23 pathway blockade reduces Th17 inflammation; direct senescence data limited—plausible indirect reduction of inflammatory SASP (Guo et al. 2023)
Kinase modulators	Imatinib	BCR‐ABL, PDGFR	Inhibits PDGF‐driven angiogenesis/fibrosis (Dhiman et al. 2020)	Direct/Context‐dependent induction. Several studies report imatinib can induce senescence markers in tumor cell lines (Drullion et al. 2012)
Kinase modulators	Sunitinib	VEGFR, multikinases	Multi‐kinase anti‐angiogenic with established vascular regression effects (García‐Alfonso et al. 2014)	Direct (tumor/EC context). Sunitinib induces senescence‐like phenotypes in tumor and endothelial cells (SA‐β‐gal, p53/Dec1 changes) and can provoke EC inflammatory change (Wang et al. 2020)
Kinase modulators	Palbociclib	CDK4/6	Primarily cytostatic; vascular effects are context dependent (Ruscetti et al. 2020)	Direct. Palbociclib induces durable G0/G1 arrest and senescence in multiple tumor models; can also induce senescence features in endothelial/stromal cells with paracrine SASP consequences (Jost et al. 2021)

*Note:* Evidence levels vary across agents, and for several compounds the relevance to IBD‐associated colorectal carcinogenesis is indirect or extrapolated from colorectal cancer, inflammatory, or other preclinical models.

## 6 Conclusion

This review delineates vascular aging as a pivotal interface between chronic intestinal inflammation and colorectal carcinogenesis in IBD. Persistent senescence within the mucosal niche fuels a self‐perpetuating cycle of SASP‐driven angiogenesis, immune dysregulation, and epithelial instability, collectively sustaining tumor‐permissive conditions. The senescence–angiogenesis axis therefore represents not only a mechanistic bridge but also a therapeutic window through which disease progression may be intercepted.

Therapeutic strategies that integrate anti‐inflammatory and anti‐angiogenic regimens with senescence‐directed interventions hold promise for restoring mucosal homeostasis. Senolytics may selectively ablate senescent cells, whereas senomorphics can reprogramme the SASP landscape to mitigate vascular and inflammatory signaling. Such combination approaches could remodel the aging microenvironment, dampen angiogenic drive, and delay neoplastic evolution in high‐risk IBD populations.

Future translation requires mechanistic precision—leveraging single‐cell and spatial technologies to chart senescent, vascular interactions and the development of reliable biomarkers to guide patient stratification and therapeutic monitoring. By targeting vascular aging as a modifiable hallmark of the inflamed intestine, precision interventions may emerge to prevent colitis‐associated neoplasia and improve long‐term outcomes in IBD.

## Author Contributions

Ruoshu Duan led the literature search and manuscript drafting. Qingyu Chen contributed to literature collection, selected drafting, and figure/table preparation. Yuan Xu assisted with literature organization and revision. Ye Yan contributed to conceptual design and critical revision. Sujing Jiang supervised the review, provided overall guidance, and finalized the manuscript. All authors read and approved the final manuscript.

## Funding

The authors have nothing to report.

## Ethics Statement

The authors have nothing to report.

## Conflicts of Interest

The authors declare no conflicts of interest.

## Data Availability

The authors have nothing to report.
